# Centrosomal Localisation of the Cancer/Testis (CT) Antigens NY-ESO-1 and MAGE-C1 Is Regulated by Proteasome Activity in Tumour Cells

**DOI:** 10.1371/journal.pone.0083212

**Published:** 2013-12-10

**Authors:** Anna Pagotto, Otavia L. Caballero, Norbert Volkmar, Sylvie Devalle, Andrew J. G. Simpson, Xin Lu, John C. Christianson

**Affiliations:** 1 Ludwig Institute for Cancer Research, Nuffield Department of Clinical Medicine, University of Oxford, Headington, Oxford, United Kingdom; 2 Ludwig Collaborative Group, Department of Neurosurgery, Johns Hopkins University School of Medicine, Baltimore, Maryland, United States of America; 3 Ludwig Institute for Cancer Research, New York Branch at Memorial Sloan-Kettering Cancer Center, New York, New York, United States of America; Mie University Graduate School of Medicine, Japan

## Abstract

The Cancer/Testis (CT) antigen family of genes are transcriptionally repressed in most human tissues but are atypically re-expressed in many malignant tumour types. Their restricted expression profile makes CT antigens ideal targets for cancer immunotherapy. As little is known about whether CT antigens may be regulated by post-translational processing, we investigated the mechanisms governing degradation of NY-ESO-1 and MAGE-C1 in selected cancer cell lines. Inhibitors of proteasome-mediated degradation induced the partitioning of NY-ESO-1 and MAGE-C1 into a detergent insoluble fraction. Moreover, this treatment also resulted in increased localisation of NY-ESO-1 and MAGE-C1 at the centrosome. Despite their interaction, relocation of either NY-ESO-1 or MAGE-C1 to the centrosome could occur independently of each other. Using a series of truncated fragments, the regions corresponding to NY-ESO-1_91-150_ and MAGE-C1_900-1116_ were established as important for controlling both stability and localisation of these CT antigens. Our findings demonstrate that the steady state levels of NY-ESO-1 and MAGE-C1 are regulated by proteasomal degradation and that both behave as aggregation-prone proteins upon accumulation. With proteasome inhibitors being increasingly used as front-line treatment in cancer, these data raise issues about CT antigen processing for antigenic presentation and therefore immunogenicity in cancer patients.

## Introduction

Engaging the immune system to recognise and eliminate tumours/cancer cells remains a promising therapeutic strategy for cancer treatment. The approach inherently relies on identification of molecular signatures able to effectively and consistently differentiate the malignant population. The Cancer/Testis (CT) antigens are a collection of more than 100 gene families with multiple members and splicing variants [1-3] that have been identified through a wide range of techniques including: T-cell epitope cloning [4-7]; serological analysis of cDNA expression libraries (SEREX) [1], differential gene expression analysis [8,9]; and bioinformatics methods [10,11]. Their expression is ordinarily restricted to the germ cells of testis [12-15] and occasionally ovary [16] and trophoblasts [17]. However, in a variety of tumour types (e.g. melanoma, small cell lung cancer, sarcoma, etc…) atypical expression of one or more CT antigens can be observed [3,18,19]. The physiological consequences of CT antigen expression for cancer progression are not fully understood, but several CT antigens have been shown to be modulators of ubiquitination through complexes formed with RING-type ubiquitin ligases [20]. 

The CT antigen NY-ESO-1/CTAG1/CT6 was first identified by SEREX in oesophageal squamous cell carcinoma [1,21]. NY-ESO-1 exhibits a relatively unique architecture, with a Pcc-1 domain in the C-terminus (aa 89-164) homologous to a yeast transcription factor involved in cell cycle progression and polarised growth [22], being its only conserved feature. A definitive biological role for NY-ESO-1 remains undetermined, but it has been shown to interact specifically with another CT antigen, MAGE-C1 [23]. MAGE-C1 is part of the larger *MAGE* (Melanoma Antigen Genes) family, which is comprised of more than 50 genes in multiple subfamilies (*MAGE-A to –L*). The predominant feature of these families is the aptly named MAGE homology domain (MHD), a large central region conserved across its members [24-26]. The MHD is present in most metazoan MAGE proteins, but notably absent in *C. elegans* as well as unicellular eukaryotes. Identified by SEREX and representational difference analysis (RDA) [8], MAGE-C1/CT7 is almost three times larger than any other MAGE family member (1142 aa). Its extended N-terminus has little to no appreciable predicted domain architecture, apart from multiple repeat sequences of 14, 16 and 21 aa [8]. MAGE-C1 is commonly expressed in multiple myeloma (MM) [27], as well as sarcoma, melanoma and bladder cancer [3,18]. A function for MAGE-C1 has yet to be determined but several studies have linked it with apoptosis in MM [28,29]. 

Among the CT antigen gene families, at least 19 members have been found to elicit humoral and/or cellular immune responses in cancer patients [19,30]. CT antigen proteins processed into peptides by the proteasome and presented on the cell surface by MHC molecules, are recognised by autologous cytotoxic T lymphocytes. Tumour-restricted expression and high immunogenicity has made CT antigens attractive targets for immunotherapeutic strategies in the treatment of selected cancers [19,31-36]. NY-ESO-1 is considered to be one of the most immunogenic CT antigens and has been a focus of investigation for the formulation of therapeutic vaccines [37]. Unlike other antigens, it is common to observe simultaneous antibody and T-cell response to NY-ESO-1, which is able to elicit strong integrated CD4^+^ and CD8^+^ T cell immune response [38-40]. Systematic analysis has identified an epitope “hot spot” for the T-cell response in the central portion of the NY-ESO-1 protein between amino acids 80-110 [41-44]. 

While transcriptional regulation of CT antigen expression has garnered much of the attention, understanding their post-translational regulation and biological function must also be considered to delineate their roles in cancer. As attractive vaccine targets, determining cellular mechanisms that control NY-ESO-1 and MAGE-C1 steady-state protein levels is important, as it may provide insight into means that could modulate their expression or processing for antigen presentation and consequently, the immune response against the tumour cells expressing these CT antigens. Here, we provide evidence that steady-state levels, solubility and localisation of the CT antigens NY-ESO-1 and MAGE-C1 are regulated by degradation through the proteasome. The specific domains of each protein involved in regulating these facets are also identified and discussed. 

## Results

### Proteasome inhibition induces insolubility and centrosomal enrichment of NY-ESO-1

**Figure 1:**
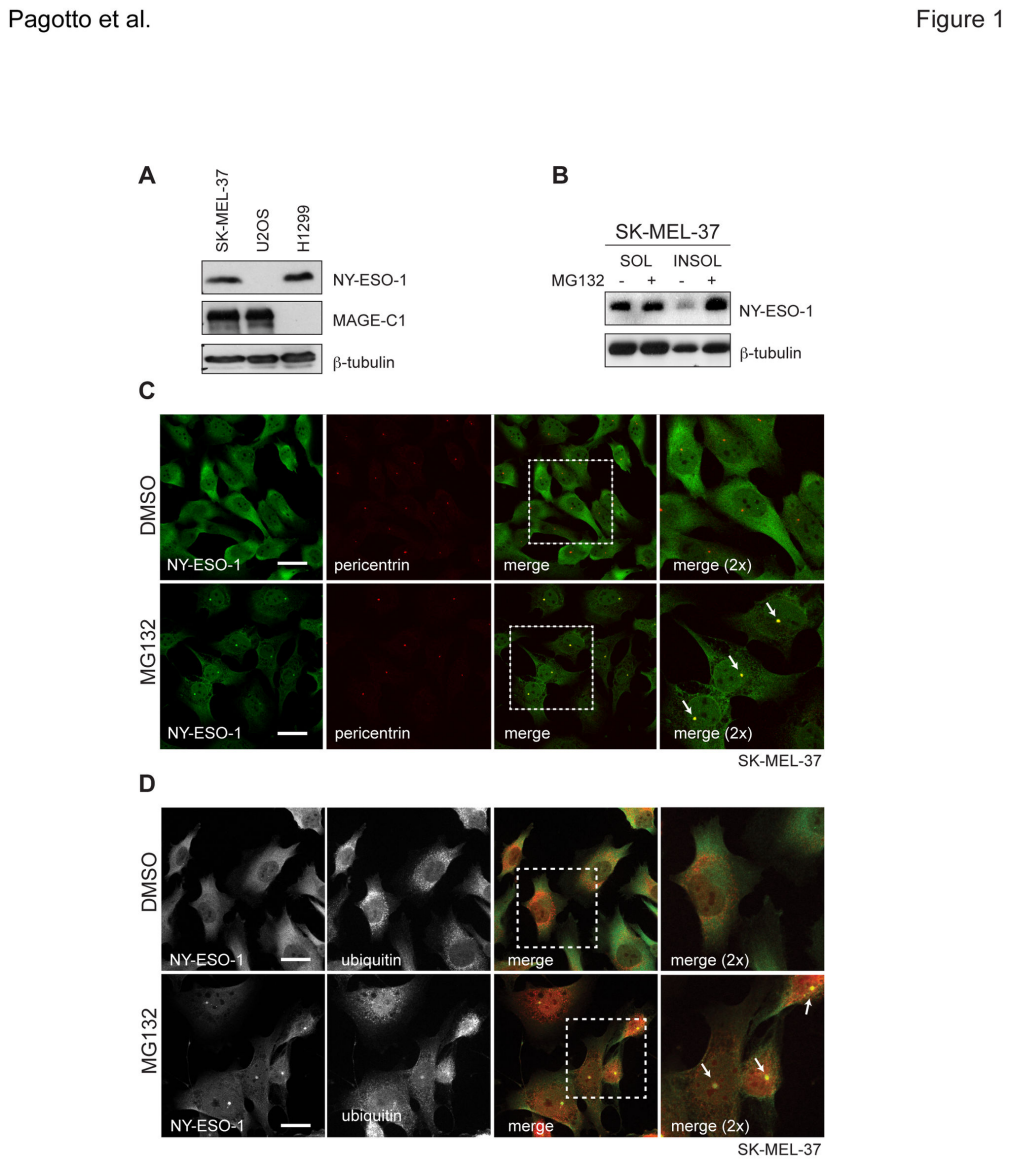
NY-ESO-1 accumulates at centrosomes upon inhibition of proteasome activity. **A**) Detection of endogenous MAGE-C1 and NY-ESO-1 by western blot (CT7.33 and NY-41 antibodies, respectively). Cell lysates were prepared from SK-MEL-37, U2OS and H1299 cells and separated by SDS-PAGE. β-tubulin served as a loading control. **B**) Detection of NY-ESO-1 from SK-MEL-37 cells treated with DMSO (negative control) and MG132 (40μM, 4hrs). Equal amounts of RIPA-soluble (SOL) and –insoluble (INSOL) material (20μg) were detected. **C**) Immunofluorescence micrographs of endogenous NY-ESO-1 (green) and pericentrin (red) in SK-MEL-37 cells are shown, along with their merged images. **D**) Immunofluorescence micrographs of endogenous NY-ESO-1 (green) and ubiquitin (red) in SK-MEL-37 cells are shown, along with their merged images. White arrows indicate centrosomes and scale bars = 20μm.

To determine whether degradation via the ubiquitin-proteasome system (UPS) may be involved in post-translational CT antigen regulation, the expression levels of NY-ESO-1 and MAGE-C1 were first determined in a panel of cancer cell lines. We observed that SK-MEL-37 melanoma and H146 small cell lung carcinoma (SCLC) cell lines express high levels of NY-ESO-1 (Figure 1A and S1A, respectively). Both cell lines were subsequently treated with the proteasome inhibitor MG132. Cell lysates were separated into soluble (SOL) and insoluble (INSOL) fractions in RIPA lysis buffer, collected and separated by SDS-PAGE. Although not quantitative, western blots probed for NY-ESO-1 detected elevated levels in the RIPA-insoluble fractions of both cell lines following MG132 treatment when compared to untreated (Figure 1B, S1B), reflecting a decreased solubility with the loss of proteasome activity. With this change in solubility, it was suspected that NY-ESO-1 localisation might have also been altered. In both SK-MEL-37 and H146 cells, NY-ESO-1 was observed by immunofluorescence to accumulate at single puncta following MG132 treatment, in addition to the predominantly cytoplasmic localisation seen in untreated cells. Reminiscent of the centrosome, NY-ESO-1’s presence at this body was confirmed by colocalisation of NY-ESO-1 with pericentrin, a well-characterised protein that marks this structure (Figure 1C, S1C). Moreover, ubiquitin puncta, a hallmark of aggresome formation, were also observed to colocalise with NY-ESO-1 at this structure (Figure 1D). With some frequency, centrosome-localised NY-ESO-1 could even be detected in untreated H146 cells (Figure S1C, top, 2D), raising the possibility of a bona fide physiological function at this body under normal conditions. These data indicate NY-ESO-1 can localise to the centrosome and that it requires the UPS for efficient degradation. 

### NY-ESO-1 and MAGE-C1 colocalise at the centrosome upon proteasome inhibition

**Figure 2:**
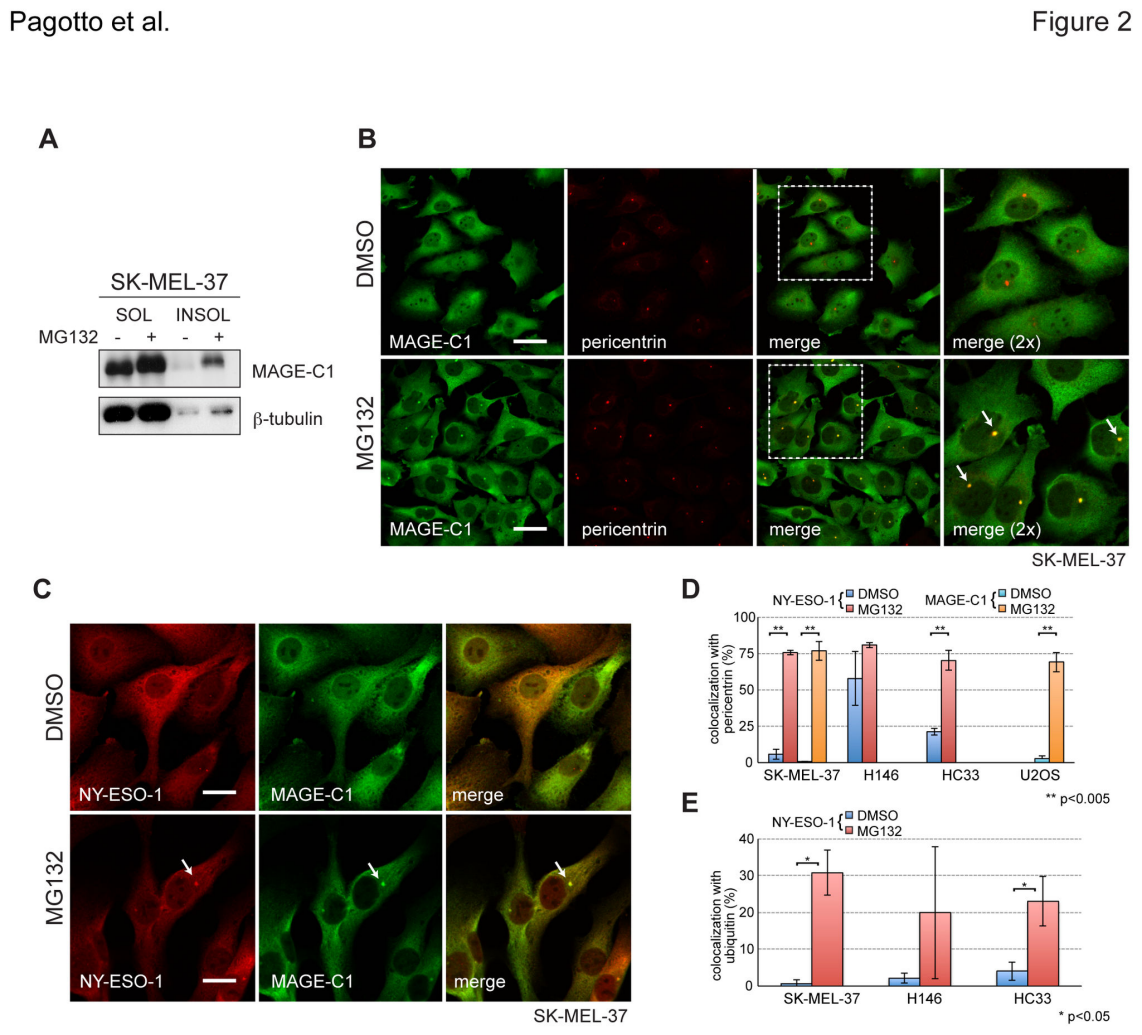
MAGE-C1 enriches at centrosomes with inhibition of proteasome activity. **A**) Western blot of MAGE-C1 from lysates of SK-MEL-37 cells treated with DMSO (negative control) and MG132 (40μM, 4hrs). RIPA-soluble (SOL) and –insoluble (INSOL) fractions are shown. **B**) Immunofluorescence micrographs of endogenous MAGE-C1 (green) and pericentrin (red) in SK-MEL-37 cells. Merged images are also shown. Scale bars = 20μm C) Immunofluorescence micrographs of endogenous MAGE-C1 (green) and NY-ESO-1 (red) in SK-MEL-37 cells. Scale bars = 10μm. In all images, white arrows indicate centrosomes. **D**) Bar graphs presenting the percentage of NY-ESO-1 and MAGE-C1 puncta colocalised with pericentrin for SK-MEL-37 (MAGE-C1/pericentrin - DMSO: 0.42±0.72 and MG132: 76.94±6.61, p=0.00222; NY-ESO-1/pericentrin – DMSO: 5.77±3.74 and MG132: 75.77±1.73, p=0.00013), U2OS (MAGE-C1/pericentrin - DMSO: 2.75±1.74 and MG132: 69.28±6.79, p=0.00212), HC33 (NY-ESO-1/pericentrin - DMSO: 21.32±2.39 and MG132: 70.23±6.86, p=0.00312) and H146 (NY-ESO-1/pericentrin - DMSO: 57.86±18.53 and MG132: 80.95±1.80, p=0.16289) cells. Although H146 cells express MAGE-C1, it was not included in this analysis. **E**) Bar graphs presenting the percentage of colocalised NY-ESO-1 and ubiquitin puncta for SK-MEL-37 (DMSO: 0.65±1.12 and MG132: 30.81±6.09, p=0.01139), HC33 (DMSO: 4.09±2.52 and MG132: 23.06±6.70, p=0.02739), and H146 (DMSO: 2.14±1.43 and MG132: 20.00±18.03, p=0.22733) cells. Mean, standard deviation and significance are shown.

NY-ESO-1 is frequently co-expressed with MAGE-C1 in cancer cells where they are able to interact. To determine whether changes to NY-ESO-1 solubility and subcellular localisation induced by MG132 treatment also affected MAGE-C1, SK-MEL-37 cells that endogenously express both MAGE-C1 and NY-ESO-1 (Figure 1A) were treated with MG132. Like NY-ESO-1, inhibiting proteasome activity markedly elevated MAGE-C1 protein levels in the RIPA-insoluble fraction, with very little change observed in the RIPA-soluble fraction (Figure 2A). Colocalisation with pericentrin demonstrated that, like NY-ESO-1, MAGE-C1 accumulates at centrosomes when proteasome activity is compromised (Figure 2B) and colocalisation of MAGE-C1 and NY-ESO-1 in similar puncta supports their simultaneous presence there (Figure 2C). Colocalisation with pericentrin increased significantly in SK-MEL-37 cells treated with MG132, reaching upwards of 75% for both MAGE-C1 (p=0.00222, 2-tailed t-test) and NY-ESO-1 (p=0.00013) (Figure 2D) and indicating a robust response to proteasome inhibition. NY-ESO-1 colocalisation with ubiquitin puncta showed a marked difference in SK-MEL-37 cells (p=0.01139) but not H146s, which exhibited less consistent formation (Figure 2E). SK-MEL-37 cells treated with either epoxomicin, a more selective proteasome inhibitor (Figure S2A), or reduced MG132 concentrations (Figure S2B) yielded comparable results. Accumulation of either MAGE-C1 or NY-ESO-1 at puncta and in RIPA-insoluble fractions was reversible, reflected by a return to dispersed localisation and detergent solubility following MG132 washout (Figure S2C, S2D). Together, these data indicate that both NY-ESO-1 and MAGE-C1 transiently accumulate at centrosomes in response to an impaired degradation pathway.

### Independent localisation of CT antigens at the centrosome

**Figure 3:**
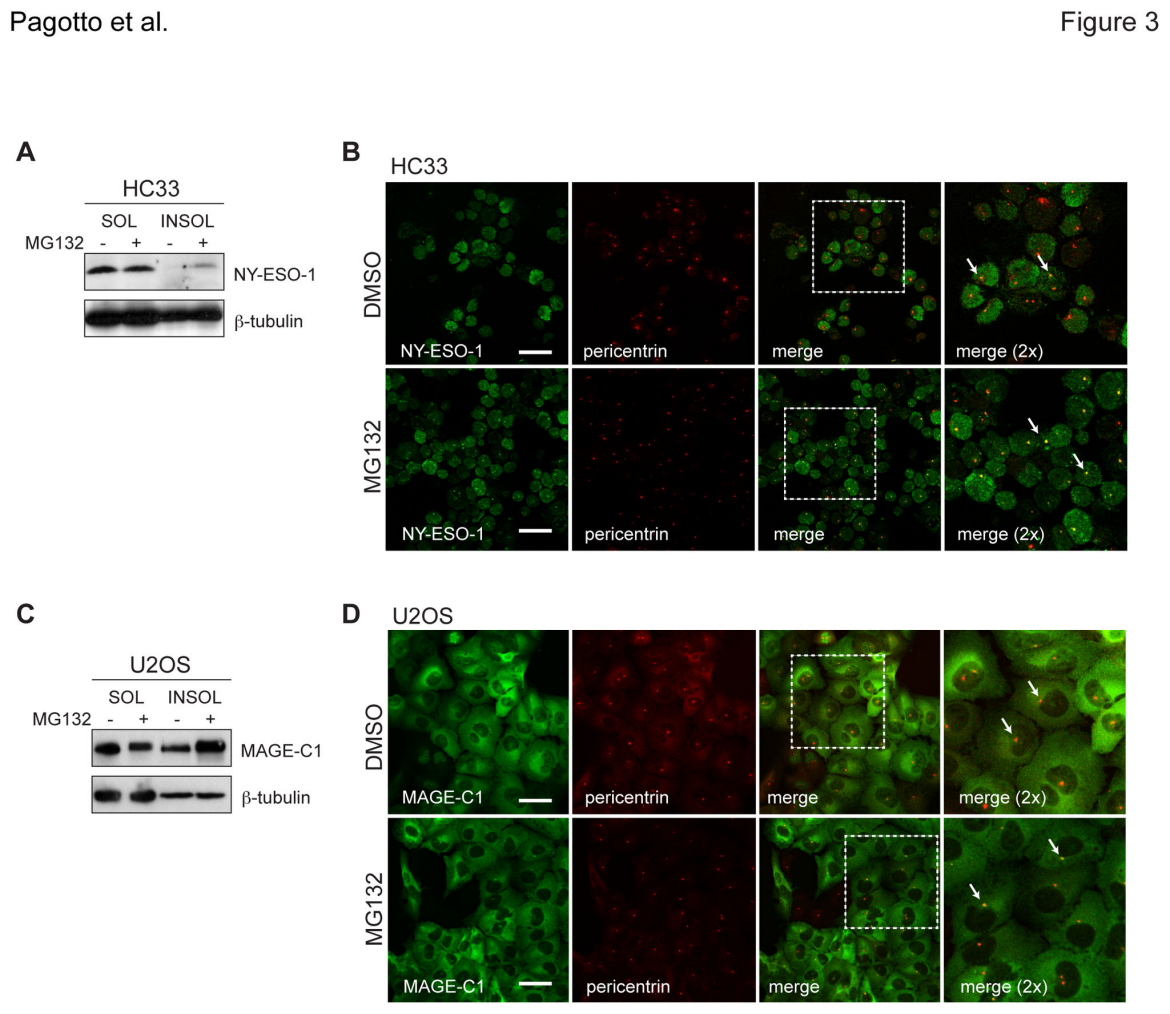
NY-ESO-1 and MAGE-C1 independently localise at centrosomes with proteasome impairment. **A**) Detection of endogenous NY-ESO-1 by western blot from HC33 SCLC cells treated with DMSO and MG132 (40μM, 4hrs). RIPA-soluble (SOL) and –insoluble (INSOL) fractions are shown. **B**) Immunofluorescence micrographs of endogenous NY-ESO-1 (green) and pericentrin (red) in HC33 cells treated with DMSO and MG132, as in Figure 1C **C**) Detection of endogenous MAGE-C1 from U2OS osteosarcoma cells under the same conditions as in Figure 2A. **D**) Immunofluorescence micrographs of endogenous MAGE-C1 (green) and pericentrin (red) in U2OS cells treated with DMSO and MG132, as in Figure 2B. White arrows indicate centrosomes. Scale bars = 20μm.

Since NY-ESO-1 and MAGE-C1 can form complexes, we next tested whether the interaction was required to confer partitioning to a RIPA-insoluble fraction or localisation at the centrosome upon proteasome inhibition. U2OS osteosarcoma cells express MAGE-C1 but not NY-ESO-1, while HC33 SCLC cells express NY-ESO-1 but not MAGE-C1 (Figure 1A, S1A). Upon treatment with MG132, each CT antigen was observed to independently accumulate in RIPA-insoluble fractions (Figure 3A, 3C) and localise to centrosomes (Figure 3B, 3D). These data were supported by siRNA-mediated gene silencing of either antigen in SK-MEL-37 cells, which demonstrated that depletion of NY-ESO-1 did not affect centrosomal localisation of MAGE-C1 (Figure S3, left) and vice versa (Figure S3, right). Both MAGE-C1 and NY-ESO-1 formed puncta that colocalised with pericentrin in U2OS (p=0.00212) and HC33 (p=0.00312) cells, respectively, with levels near 70% and significantly greater than DMSO-treated cells (Figure 2D). Like the H146s, higher basal levels of NY-ESO-1 puncta were also observed in the HC33 cell line. Collectively, these data indicate that NY-ESO-1 and MAGE-C1 can accumulate and localise to the centrosome independently of each other during times of impaired proteasome activity. 

### NY-ESO-1_91-150_ contributes to overall stability

**Figure 4:**
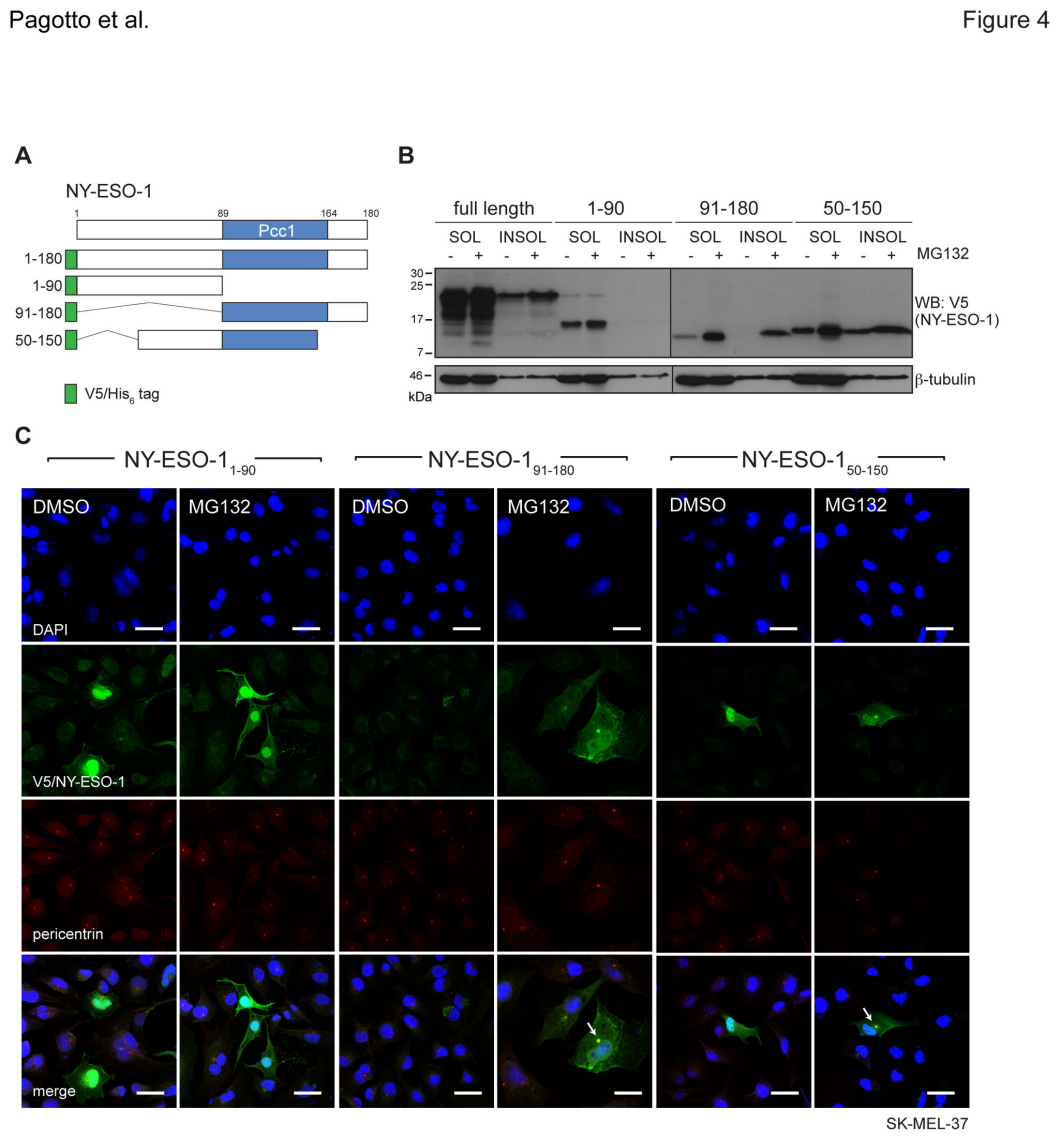
An internal region of NY-ESO-1 (aa 91-150) is sufficient for centrosome localisation after MG132 treatment. **A**) Schematic representation of V5/His6 epitope tagged full length and truncation constructs of NY-ESO-1. NY-ESO-1_1-90_, NY-ESO-1_91-180_ and NY-ESO-1_50-150_ are shown, along with the conserved Pcc1 domain. **B**) Detection of NY-ESO-1 constructs transiently expressed in NIH3T3 cells by western blot with anti-V5 and anti-β-tubulin (loading control). Cells were treated with MG132 and fractions collected as in Figure 1B. **C**) Immunofluorescence micrographs of SK-MEL-37 cells (±MG132) transiently expressing NY-ESO-1 fragments (anti-V5, green) and pericentrin (red), with nuclei identified by DAPI staining (blue). White arrows indicate centrosomes. Scale bars = 20μm.

We have demonstrated that NY-ESO-1 is a substrate of the proteasome but which regions influence its degradation is not known. To identify the domains responsible for partitioning in RIPA-insoluble fractions and centrosome localisation, we constructed fragments of NY-ESO-1 (NY-ESO-1_1-90_, NY-ESO-1_91-180_ and NY-ESO-1_50-150_, Figure 4A) and expressed them individually along with the full-length form (NY-ESO-1_FL_) in mouse derived NIH3T3 cells, which express neither MAGE-C1 nor NY-ESO-1. Following the MG132 treatment and solubilisation regimen described above, relative expression and subcellular localisation were assayed by western blot and immunofluorescence, respectively. Both RIPA-soluble and -insoluble fractions of NY-ESO-1_FL_, NY-ESO-1_91-180_ and NY-ESO-1_50-150_ were stabilised by MG132, while NY-ESO-1_1-90_ only accumulated in the RIPA-soluble fraction (Figure 4B). Marked accumulation of the NY-ESO-1_91-180_ fragment with MG132 implicates the UPS in the fragment’s rapid turnover and suggests NY-ESO-1 stability is derived from a region outside the Pcc1 domain. The NY-ESO-1_1-90_ fragment appeared concentrated in the nucleus, while NY-ESO-1_91-180_ and NY-ESO-1_50-150_ localised to both the nuclear and cytoplasmic regions regardless of whether endogenous NY-ESO-1 and MAGE-C1 were present (SK-MEL-37, Figure 4C) or not (NIH3T3, Figure S4). Subcellular localisation of either NY-ESO-1 truncations expressed in NIH3T3s (Figure S4) or NY-ESO-1_1-90_ in SK-MEL-37s (Figure 4C) was not markedly altered by MG132. Yet like endogenous NY-ESO-1, both NY-ESO-1_91-180_ and NY-ESO-1_50-150_ accumulated at centrosomes in SK-MEL-37 cells (Figure 4C). Together, these data highlight the region between aa 91-150 as influential in NY-ESO-1 stability and in localisation at the centrosome.

### The MHD contributes to MAGE-C1 stability

**Figure 5:**
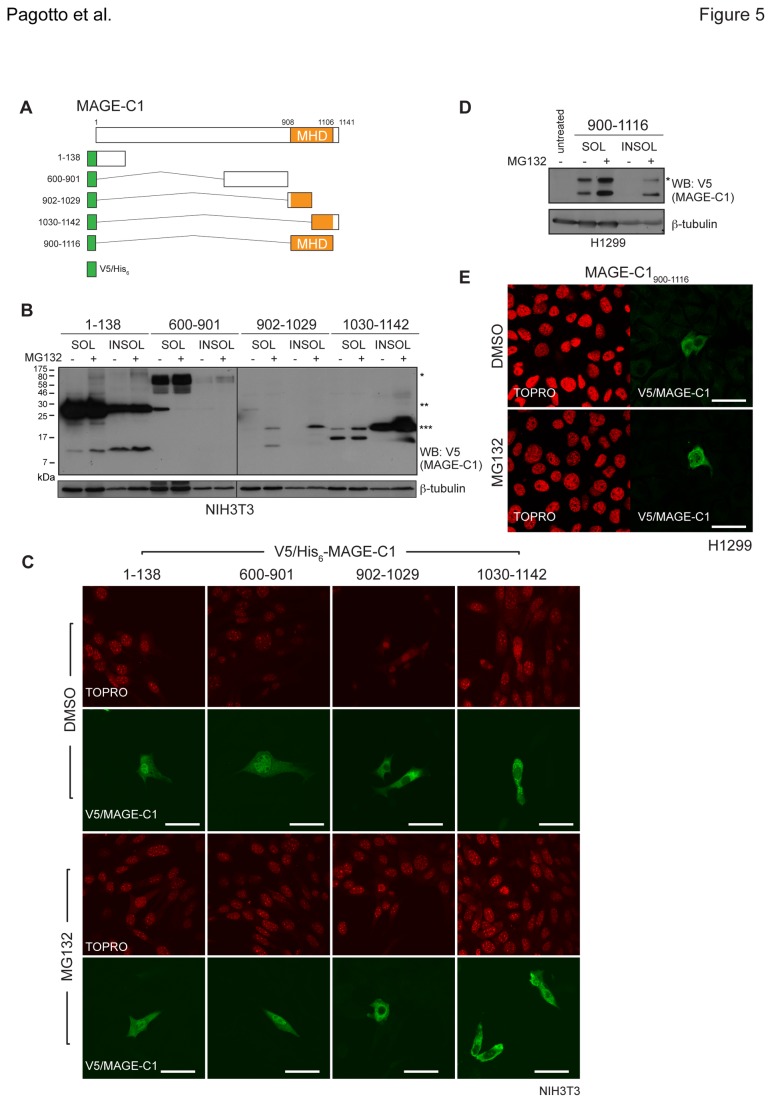
The MAGE homology domain is important for MAGE-C1 localisation and solubility. **A**) Schematic representations of V5/His6 epitope tagged full length and truncation constructs of MAGE-C1. MAGE-C1_1-138,_ MAGE-C1_600-901_, MAGE-C1_902-1029_, MAGE-C1_1030-1142_ and MAGE-C1_900-1116_ are shown and the MAGE homology domain (MHD) is indicated. **B**) Transient expression of MAGE-C1 fragments in NIH3T3 mouse fibroblasts detected by western blot with anti-V5 and anti-β-tubulin (loading control). Cells were treated and lysed as in Figure 1B. Asterisks indicate possible oligomeric forms. **C**) Immunofluorescence micrographs of NIH3T3 mouse fibroblasts expressing MAGE-C1 fragments (anti-V5, green) where TOPRO was included to stain nuclei (red). Cells were treated with MG132 (40µM, 4hr) or DMSO (negative control). **D**) Transient expression of MAGE-C1_900-1116_ in H1299 NSCLC cells and detected under the conditions used in Figure 5B. Asterisk indicates possible oligomeric form. **E**) Immunofluorescence micrographs of H1299 cells expressing MAGE-C1_900-1116_ as in Figure 5C. For all images, scale bars = 20μm.

Like NY-ESO-1, the aspects impacting MAGE-C1 steady state levels are not well characterised. To determine the region/s of MAGE-C1 influencing its stability, we generated a series of fragments including: MAGE-C1_1-138_, MAGE-C1_600-901_, MAGE-C1_902-1029_ and MAGE-C1_1030-1142_ (Figure 5A). We were unable to construct fragments spanning the entire length of MAGE-C1, as the high number of GC-rich repeats between amino acids 139 and 600 precluded amplification of this region. When transiently transfected into both NIH3T3 (Figure 5B) and H1299 cells (Figure S5), MAGE-C1_1-138_ and MAGE-C1_600-901_ displayed higher steady-state levels of predominantly single bands, compared to other truncations. Moreover, each appeared to migrate at a molecular weight that was higher than expected. This could represent the formation of MAGE-C1 oligomers or could reflect retarded migration owing to polypeptide composition. In contrast, both C-terminal fragments (MAGE-C1_902-1029_ and MAGE-C1_1030-1142_) exhibited reduced RIPA-soluble steady-state levels in which two distinct bands were detected. The higher than expected bands may reflect a post-translational modification (e.g. ubiquitination) or potentially an oligomeric species. Fragments containing portions of the MHD (MAGE-C1_902-1029_, MAGE-C1_1030-1142_) accumulated in both the RIPA-soluble and –insoluble fractions with addition of MG132. Conversely, MAGE-C1_600-901_ was only modestly stabilised with MG132 while the N-terminal fragment (MAGE-C1_1-138_) appeared to be largely unaffected (Figure 5B). In NIH3T3 cells, MAGE-C1_1-138_ and MAGE-C1_600-901_ appeared in both the nucleus and cytoplasm, while the MHD-containing fragments (MAGE-C1_902-1029_ and MAGE-C1_1030-1142_) were entirely excluded from the nucleus (Figure 5C). Localisation of the MAGE-C1 fragments in NIH3T3 cells was not markedly altered following MG132 treatment. To determine whether the MHD alone was sufficient for MAGE-C1 partitioning and localisation, the isolated domain (MAGE-C1_900-1116_) was expressed in H1299 cells, a human lung carcinoma cell line that expresses NY-ESO-1 but not MAGE-C1. Like the fragments that contained portions of the MHD, MAGE-C1_900-1116_ accumulated in both RIPA-soluble and-insoluble fractions with MG132 as two immunoreactive bands detected by western blot (Figure 5D). Consistent with the related fragments, MAGE-C1_900-1116_ was predominantly cytoplasmic in H1299 cells and MG132 did not notably alter that localisation (Figure 5E). These data implicate the highly conserved MAGE homology domain in the proteasome-dependent processing and cytoplasmic localisation of MAGE-C1. 

## Discussion

Immunotherapeutic strategies for cancer treatment rely on effectively discriminating between normal and malignant cells based on differential expression patterns of cell surface proteins or presented antigens. The Cancer/Testis (CT) antigen family of genes has over 100 different members, whose normally restricted expression in germ line cells of the testis becomes dysregulated in many cancers, effectively differentiating them from the normal, surrounding tissue. CT antigen genes are normally silenced by methylation on their CpG rich promoters and so demethylation of such regions, as well as histone acetylation, is partially responsible for their aberrant re-expression in cancer (reviewed in 19). However, these mechanisms are unable to entirely explain the lack of expression of CT antigens in some tumour types characterised by global hypomethylation (e.g. colon cancer) [45,46] nor the heterogeneity often observed in tumour samples [47]. Such complex expression patterns require multiple levels of regulation that are unlikely to be attributable solely to transcriptional mechanisms. For this reason, we investigated the understudied post-translational regulation of CT antigens. Here we present data implicating degradation, via the 26S proteasome, as an important regulator of CT antigen steady state levels and demonstrate that when it is compromised, solubility and subcellular localisation of both NY-ESO-1 and MAGE-C1 are dramatically altered. 

 The ubiquitin-proteasome system (UPS) is a principal mechanism for regulated protein degradation in eukaryotes (reviewed in 48). To ascertain how degradation via the UPS impacted steady-state levels of NY-ESO-1 and MAGE-C1 in cancer cell lines, we treated cells with proteasome inhibitors while monitoring changes to solubility and localisation of each protein. Proteasome inhibitors caused endogenously expressed NY-ESO-1 and MAGE-C1 to accumulate, either alone (HC33, U2OS), when both CT antigens were present (SK-MEL-37) or when each was transiently expressed in cell lines lacking either (NIH3T3). Of note, accumulation was predominantly detected as an increase in the detergent-insoluble fraction of the cell lysate. While neither NY-ESO-1 nor MAGE-C1 has been reported previously to be aggregation-prone, the propensity for CT antigens to become insoluble suggests that the capacities of the chaperone (and degradation) network/s have been exceeded. Both MAGE-C1 and NY-ESO-1 were observed to accumulate at the centrosome under the same conditions. This pattern is reminiscent of other aggregation prone proteins such as CFTRΔF508 [49] and huntingtin [50] whose accumulation at centrosomes is exacerbated with proteasome inhibition (reviewed in 51,52). Centrosomes serve as the primary microtubule organising center (MTOC) and play an important role as a regulator of cell-cycle progression [53]. The MTOC also becomes a hub for components of both cytosolic chaperones (e.g. Hsp40, Hsp70) and the UPS (e.g. ubiquitin, 19S and 20S subunits) [49,54,55], which accumulate in response to misfolded proteins delivered there by direct transport on microtubules to form the intracellular body referred to as the aggresome [49,56,57]. In this role, centrosomes function as triage platforms for protein quality control decisions. There is evidence that the centrosome-localised UPS components are able to engage in active degradation [54,58,59]. The insoluble partitioning and accumulation at centrosomes of both NY-ESO-1 and MAGE-C1 suggest a propensity of these CT antigens to aggregate, a feature exacerbated when proteasome activity is compromised. 

 Accumulation of both NY-ESO-1 and MAGE-C1 at centrosomes in the absence of proteasome activity likely reflects the segregation of misfolded proteins (possibly as insoluble aggregates) to a central location where they await remediation. The detection of concentrated ubiquitin clusters at centrosomes (Figure 1D) and the build-up of polyubiquitinated materials in the INSOL fraction (Figure S2D) support this model. Higher levels of NY-ESO-1 puncta at centrosomes in untreated H146 cells (and to a lesser extent HC33s) versus SK-MEL-37s may also reflect differences between their chaperone and degradation networks, making aggregation more prevalent in cells with reduced capacity. An alternative explanation to this might be that either NY-ESO-1 or MAGE-C1 (or both) has a bona fide functional role at the centrosome (i.e. NY-ESO-1 in H146 cells, Figure S1C, 2D). For example, proteasome inhibitors impair turnover of centrosomal proteins, affecting both microtubule nucleation and organisation [60,61]. The presence of either MAGE-C1 or NY-ESO-1 (or both) at centrosomes could modulate degradation rates and/or stability of essential factors there. The MHDs of several MAGE family members interact with RING-type E3 ubiquitin ligases (e.g. TRIM28) and modulate ubiquitination [20]. In MM, where MAGE-C1 and NY-ESO-1 are often overexpressed, centrosome amplification is found in ~30% of cases and has been associated with overexpression of CT genes of the MAGE family [62]. Thus, a gain of function effect of NY-ESO-1 and/or MAGE-C1 at the centrosome cannot be ruled out. 

 A documented complex between NY-ESO-1 and MAGE-C1 meant that localisation of each CT antigen to centrosomes or their insolubility could have been dependent on the interaction. Yet since cell lines expressing single CT antigens still accumulated at centrosomes after MG132 treatment, an interaction does not appear to be a prerequisite. This was confirmed using SK-MEL-37 cells and CT antigen targeted RNAi individually (Figure S3) and supports a model where both NY-ESO-1 and MAGE-C1 are intrinsically capable of independent centrosomal localisation. Moreover, the regions governing this localisation could be mapped to aa 91-150 of NY-ESO-1 and aa 900-1116 of MAGE-C1. This region of NY-ESO-1 is part of a ‘hot spot’, identified roughly between amino acids 80-110, for the cellular immune response against NY-ESO-1 in cancer [37,63]. Since these MHC Class I presented epitopes are the by-products of proteasomal degradation, identifying this region as important for turnover and localisation upon MG132 treatment is consistent. The MHD (aa 908-1106) of MAGE-C1 is conserved within the MAGE family but exhibits little homology with other known domains. Even though its function is still not well understood, the MHD for some MAGEs represents a site of protein-protein interaction [20,23]. In fact, MAGE-C1 appears to bind NY-ESO-1 through its MHD [19,23,30]. Two MAGE-C1 epitopes presented by MHC-I molecules on MM cells are able to induce CD8+ T-cell response [64] and those peptides are derived from the MHD (aa 959-968 and 1083-1091). The common presence of the MHD in all MAGE family members suggests that this feature of MAGE-C1 may be relevant for understanding the immunological response to other MAGE family proteins.

 Overall, our findings highlight a post-translational level of regulation of NY-ESO-1 and MAGE-C1 that may affect the immune response against CT antigen-expressing tumour cells. These observations are particularly relevant for MM. The use of selective proteasome inhibitors (e.g. Velcade/bortezomib) has been a major advancement in the treatment of MM. We have shown that proteasome inhibitors are likely to induce centrosomal localisation and accumulation of insoluble MAGE-C1 and NY-ESO-1 as part of aggresomes in MM cells. What physiological consequence CT antigen accumulation might have in MM is not yet clear. Both MAGE-C1 and NY-ESO-1 are frequently expressed in MM (66% and 22% of cases respectively), with 93% of MAGE-C1-positive myelomas eliciting a detectable humoral and cellular immune response against this antigen [65,66]. Accumulation of either CT antigen (or both) could be cytotoxic and exacerbate the resulting apoptotic program induced by proteasome inhibitors [67]. We did observe reduced MAGE-C1 staining in the nucleus when cells were treated with MG132. Myelomas with nuclear-cytoplasmic or nuclear-only MAGE-C1 have a worse prognosis when compared to those with MAGE-C1 only in the cytoplasm [27]. Although antigen presentation would be compromised, the restricted CT antigen localisation that results from proteasome inhibition may in fact positively contribute to an improved prognosis. This potential benefit must however be balanced against the well-documented pleiotropic effects on essential cellular functions that arise with proteasome inhibition. The pivotal role played by the UPS in all cells, including tumour cells, makes defining a therapeutic window key to the utility of these compounds. Further studies are needed to determine the physiological roles of CT antigens and the potential consequences to efficacy of immunotherapy against tumours that frequently express them, such as melanoma, lung carcinoma and multiple myeloma.

## Materials and Methods

### Cell culture, antibodies and reagents

Human melanoma (SK-MEL-37) [1] and small cell lung cancer (SCLC) cell lines (H146, HC33, H69, H209, H524, H889, and H2171; a kind gift of Dr. Michael Seckl and reported previously [68]), were cultured in RPMI 1640 (Gibco). U2OS osteosarcoma, NIH3T3 fibroblasts and H1299 non-small cell lung cancer (NSCLC) cells were obtained from ATCC and cultured in DMEM (Gibco). Media were supplemented with 2mM L-Glutamine, 200u/ml penicillin/streptomycin and 10% (v/v) of foetal calf serum. All cell lines were grown at 37°C in the presence of 5% CO_2_. Antibodies against the following proteins were used for western blot and immunofluorescence: pericentrin (ab28144 and ab4448, Abcam), β-tubulin (TUB2.1, Abcam), V5 (46-0705, Invitrogen), ubiquitin (P4D1, Santa Cruz and 3933, Cell Signalling Technologies) and KU80 (Ab-2, Neomarker). Anti-MAGE-C1 (CT7.33), anti-NY-ESO-1 (E978) and anti-NY-ESO-1 (NY41) have been described previously [23,69,70]. MG132 was purchased from Calbiochem and epoxomicin from Enzo Life Sciences.

### Plasmids, small interfering RNAs and transfection

V5/His_6_-tagged NY-ESO-1 (NY-ESO-1_FL,_ NY-ESO-1_1-90_, NY-ESO-1_91-180_ and NY-ESO-1_50-150_) and MAGE-C1 (MAGE-C1_1-138_, MAGE-C1_600-901_, MAGE-C1_902-1029_, MAGE-C1_1030-1142_ and MAGE-C1_MHD_) constructs were generated by PCR and subcloned into the pcDNA3.1/V5-His-TOPO vector (Invitrogen) for expression in mammalian cells. Transfections were performed using Fugene6 (Roche) according to the manufacturer’s instructions. Specific oligonucleotide sequences targeting NY-ESO-1 (5’-GGACACAGUGAACUCCUUCAGAAGCAC-3’) and MAGE-C1 (5’-GUGGAGAGGAGAGAGGGAGUCCUCCCA-3’) for gene silencing were purchased from IDT (Integrated DNA Technologies). A scrambled duplex (DS NC1) was used as a negative control. siRNAs were introduced to cells using Lipofectamine 2000 (Invitrogen) according to the manufacturer’s protocol.

### Sample preparation, SDS-PAGE and Western blotting

All cells were lysed in RIPA buffer (150 mM NaCl, 1% (v/v) NP-40, 0.1% (w/v) SDS, 50 mM Tris, pH7.4) supplemented with COmplete^TM^ protein inhibitor cocktail (Boehringer Mannheim) and iodoacetamide (5 mM, Sigma). Cell lysates were centrifuged at 14,000 rpm, 4°C for 15 min. The resulting supernatant was collected (RIPA-soluble) while the residual pellet was subsequently dissolved in an equivalent volume of Urea buffer (8M urea, 1M thiourea, 0.5% (w/v) CHAPS, 50mM DTT, 24mM spermine) for 20 min at room temperature (RT). Following a second centrifugation (20 min, 20,000 x g), the resulting supernatant was collected (RIPA-insoluble fraction). Protein concentrations were determined by Bradford assay. RIPA-soluble and –insoluble fractions were resuspended with 5x Laemmli buffer + 12.5% (v/v) β-Mercaptoethanol, boiled for 5 min and equivalent protein amounts (20μg) loaded for separation by SDS-PAGE. The resulting gels were transferred to nitrocellulose membranes for western blotting. Membranes were blocked in 1x TBS-Tween + 5% skim milk (RT, 60 min) followed by incubation with primary (RT, 3 hr or 4°C, overnight) and HRP-conjugated secondary (RT, 1 hr) antibodies in TBS-Tween + 5% skim milk. Membranes were washed in TBS-Tween (RT, 3 x 10 min) after each step. Resulting bands were detected by ECL and visualised on X-ray film (Amersham Biosciences).

### Immunofluorescence & microscopy

Cells were grown in 24 well plates on coverslips to approximately 70-80% confluence. Following treatments, cells were washed twice with PBS and fixed in 4% paraformaldehyde (RT, 20min). Cells were then permeabilised with 0.1% Triton in PBS for 4 min on ice. Samples were blocked with 0.2% fish skin gelatine (FSG, 20 min at RT). Primary antibodies were diluted in 0.2% FSG and incubated 60 min at RT. Following washes, coverslips were incubated (20 min at RT) with fluorescently labelled secondary antibodies (FITC and TRITC, Molecular Probes), diluted 1:400 in 0.2% FSG. In some cases, either TO-PRO® (Molecular Probes) or DAPI (Sigma) were included to visualise nuclei. All images were captured by confocal microscopy (Zeiss, LSM 710) and processed using LSM image viewer software.

### Statistical analyses of colocalisation

Fixed SK-MEL-37, U2OS, HC33 and H146 cells were stained for NY-ESO-1, MAGE-C1 and either pericentrin or ubiquitin (described above). The percentage of CT-antigen colocalisation was determined by comparing the number of NY-ESO-1 or MAGE-C1 puncta to the total number of pericentrin puncta or total number of cell nuclei within a field of view. Fields contained a minimum of 10 cells. Unstained cells or cells with no NY-ESO-1 or MAGE-C1 expression were not included in the analyses. Five fields of view representing technical replicates were chosen at random from each condition and the mean percentage of colocalisation determined. Mean and standard deviation for each condition in three independent experiments were then calculated (n=3) and statistical significance measured by 2-tailed t-test.

## Supporting Information

Figure S1 **Individual CT antigen expression in cancer cell lines.** **A**) Western blot analysis of endogenous MAGE-C1 and NY-ESO-1 cell lysates prepared from seven SCLC cell lines (HC33, H69, H209, H524, H2171, H146 and H889). Samples were probed for NY-ESO-1 (NY-41) and MAGE-C1 (CT7.33) with KU-80 used as a loading control. **B**) Detection of endogenous NY-ESO-1 from H146 cells treated with DMSO and MG132 (40μM, 4hrs) by western blot. RIPA-soluble (SOL) and –insoluble (INSOL) fractions are shown. **C**) Immunofluorescence micrographs of endogenous NY-ESO-1 (green) and pericentrin (red) in H146 cells treated with DMSO and MG132, as in Figure 1C. Scale bars = 20μm.
(TIF)

Figure S2 **Proteasome inhibitors cause NY-ESO-1 and MAGE-C1 to localise at centrosomes.** Immunofluorescence micrographs of endogenous MAGE-C1 and NY-ESO-1 in SK-MEL-37 cells treated with **A**) epoxomicin at 1µM and **B**) MG132 at 40µM, 20µM and 1µM. DMSO served as the negative control. Scale bars = 20μm. **C**) SK-MEL-37 cells pre-treated with 10µM MG132 (or DMSO) for 4 hrs followed by washout for 0 and 8 hrs. Immunofluorescence micrographs of endogenous MAGE-C1 (green), pericentrin (red) are shown, along with their merged images. White arrows indicate centrosomes. Scale bars = 20μm. **D**) Western blot analysis of RIPA-soluble (SOL) and –insoluble (INSOL) fractions (20μg/lane) isolated from SK-MEL-37 cells treated under the same conditions as in Figure S2C. Endogenous NY-ESO-1, MAGE-C1, polyubiquitin and a β-tubulin loading control are shown.
(TIF)

Figure S3 **RNAi of endogenous NY-ESO-1 and MAGE-C1 in SK-MEL-37 cells.** Immunofluorescence micrographs of endogenous NY-ESO-1 (red) and MAGE-C1 (green) in SK-MEL-37 cells knocked down for either NY-ESO-1 (left) or MAGE-C1 (right) by siRNA. MG132 treatment was performed as in Figure 1C. Nuclei are stained with DAPI (blue). Scale bars = 10μm.
(TIF)

Figure S4 **Localisation of transiently expressed NY-ESO-1 fragments in NIH3T3 cells.** Immunofluorescence micrographs showing NIH3T3 mouse fibroblasts expressing NY-ESO-1_1-90_, NY-ESO-1_91-180_ and NY-ESO-1_50-150_ (anti-V5, green). TOPRO was included to stain nuclei (red). Cells were treated with MG132 (40µM, 4hr) or DMSO (negative control). Scale bars = 20μm.
(TIF)

Figure S5 **Solubility of MAGE-C1 fragments transiently expressed in H1299 cells.** Transient expression of MAGE-C1 fragments in H1299 cells detected by western blot with anti-V5 and anti-β-tubulin (loading control). Cells were treated with DMSO and MG132 (40μM, 4hrs) and RIPA-soluble (SOL) and –insoluble (INSOL) fractions collected. Asterisks indicate possible oligomeric forms.
(TIF)
