# Supplementary figures and images for: Centrosomal Localisation of the Cancer/Testis (CT) Antigens NY-ESO-1 and MAGE-C1 Is Regulated by Proteasome Activity in Tumour Cells
Source: PLoS One. 2013 Dec 10;8(12):e83212. doi: 10.1371/journal.pone.0083212 (PMC3858345; doi:10.1371/journal.pone.0083212)

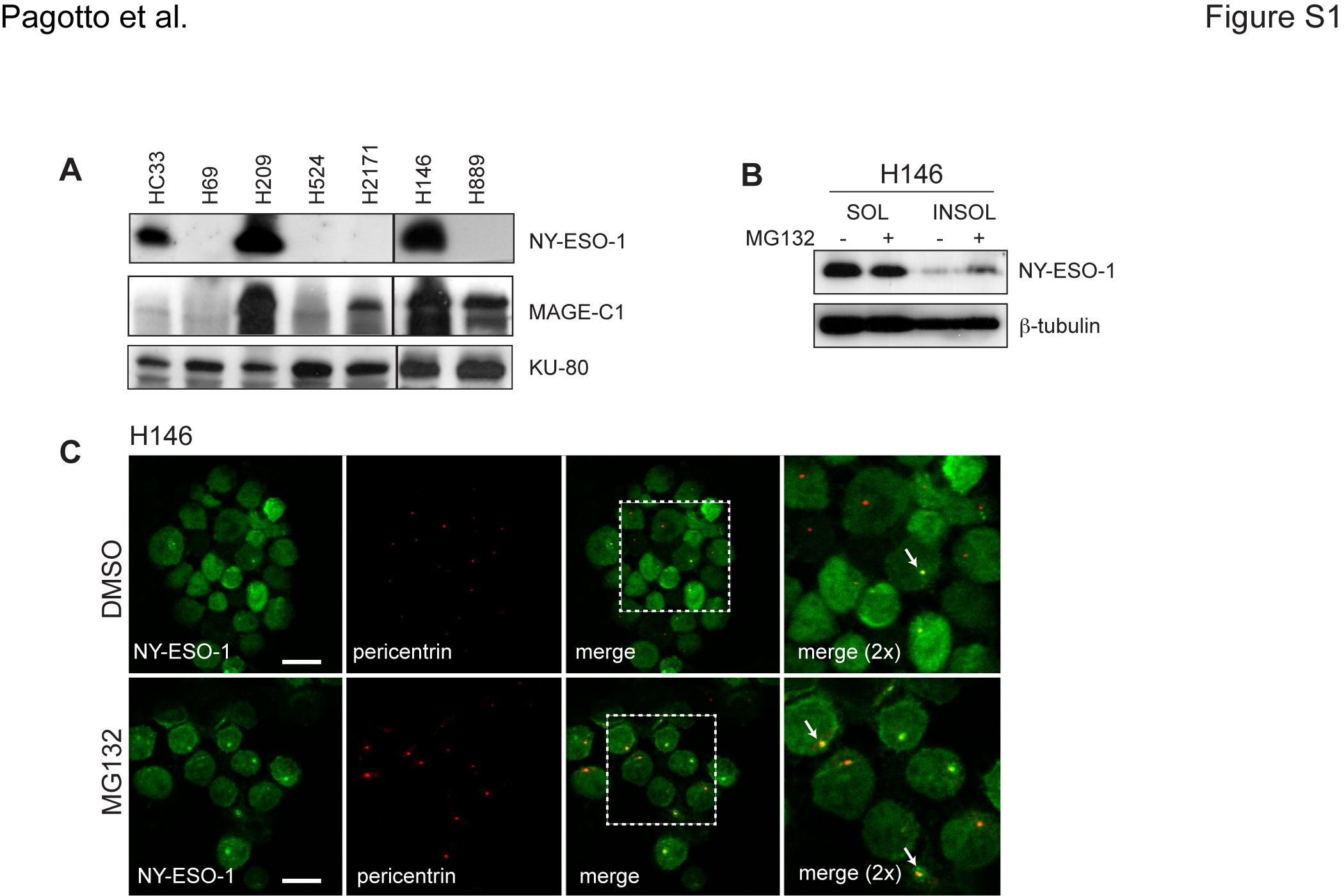

Supplement: Figure S1 — Individual CT antigen expression in cancer cell lines. A) Western blot analysis of endogenous MAGE-C1 and NY-ESO-1 cell lysates prepared from seven SCLC cell lines (HC33, H69, H209, H524, H2171, H146 and H889). Samples were probed for NY-ESO-1 (NY-41) and MAGE-C1 (CT7.33) with KU-80 used as a loading control. B) Detection of endogenous NY-ESO-1 from H146 cells treated with DMSO and MG132 (40μM, 4hrs) by western blot. RIPA-soluble (SOL) and –insoluble (INSOL) fractions are shown. C) Immunofluorescence micrographs of endogenous NY-ESO-1 (green) and pericentrin (red) in H146 cells treated with DMSO and MG132, as in Figure 1C. Scale bars = 20μm. (TIF) [file pone.0083212.s001.tif]

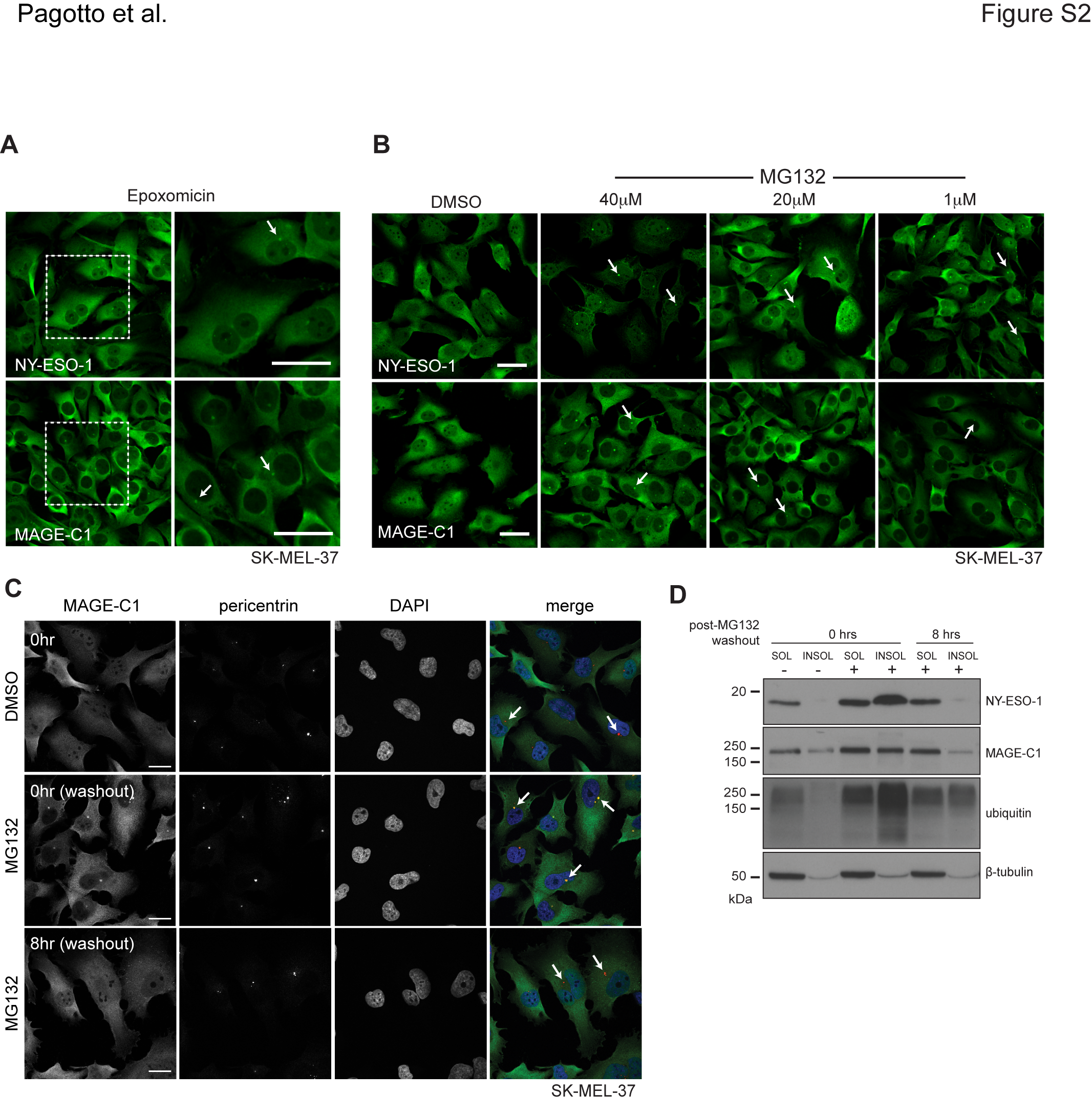

Supplement: Figure S2 — Proteasome inhibitors cause NY-ESO-1 and MAGE-C1 to localise at centrosomes. Immunofluorescence micrographs of endogenous MAGE-C1 and NY-ESO-1 in SK-MEL-37 cells treated with A) epoxomicin at 1µM and B) MG132 at 40µM, 20µM and 1µM. DMSO served as the negative control. Scale bars = 20μm. C) SK-MEL-37 cells pre-treated with 10µM MG132 (or DMSO) for 4 hrs followed by washout for 0 and 8 hrs. Immunofluorescence micrographs of endogenous MAGE-C1 (green), pericentrin (red) are shown, along with their merged images. White arrows indicate centrosomes. Scale bars = 20μm. D) Western blot analysis of RIPA-soluble (SOL) and –insoluble (INSOL) fractions (20μg/lane) isolated from SK-MEL-37 cells treated under the same conditions as in Figure S2C. Endogenous NY-ESO-1, MAGE-C1, polyubiquitin and a β-tubulin loading control are shown. (TIF) [file pone.0083212.s002.tif]

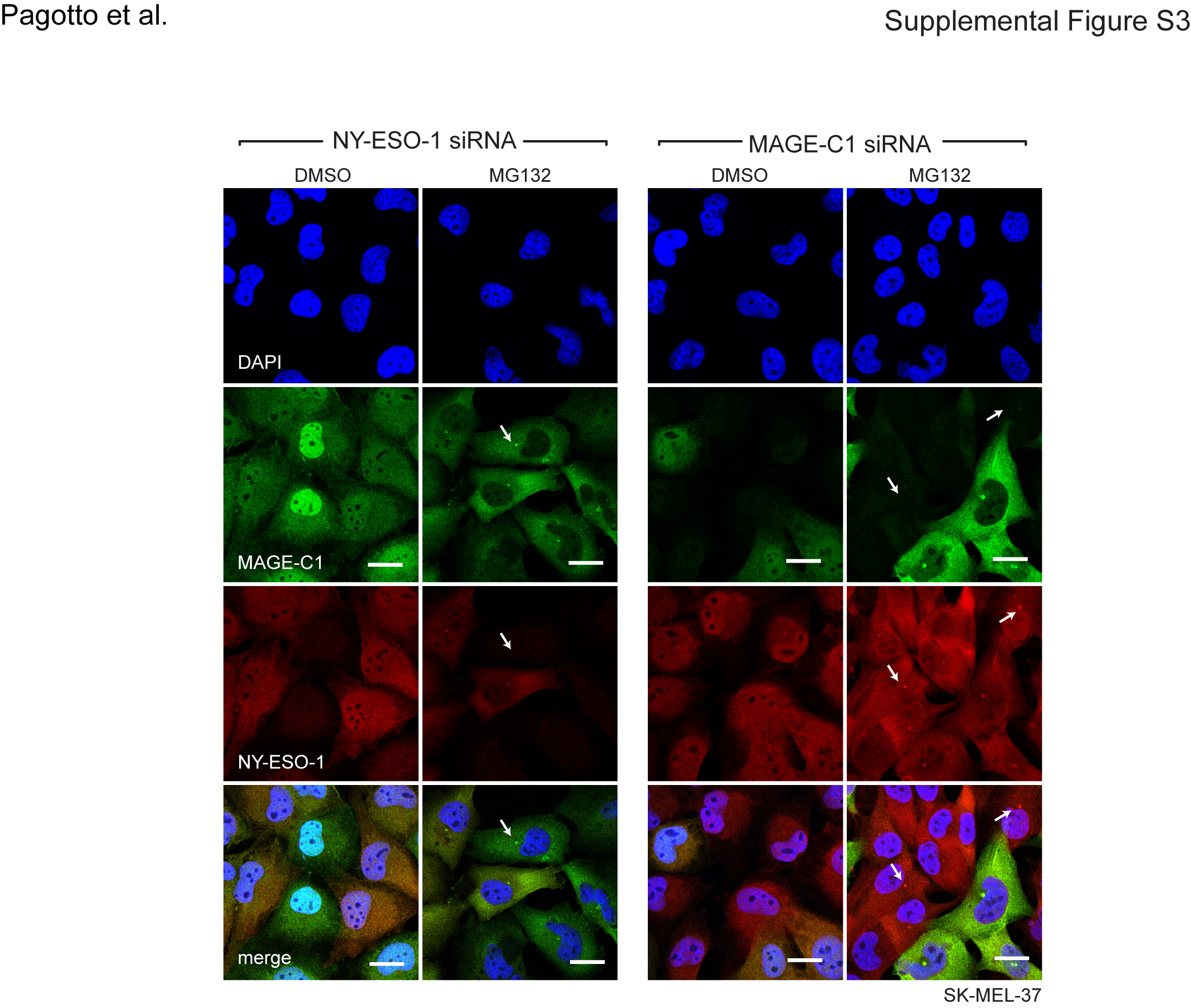

Supplement: Figure S3 — RNAi of endogenous NY-ESO-1 and MAGE-C1 in SK-MEL-37 cells. Immunofluorescence micrographs of endogenous NY-ESO-1 (red) and MAGE-C1 (green) in SK-MEL-37 cells knocked down for either NY-ESO-1 (left) or MAGE-C1 (right) by siRNA. MG132 treatment was performed as in Figure 1C. Nuclei are stained with DAPI (blue). Scale bars = 10μm. (TIF) [file pone.0083212.s003.tif]

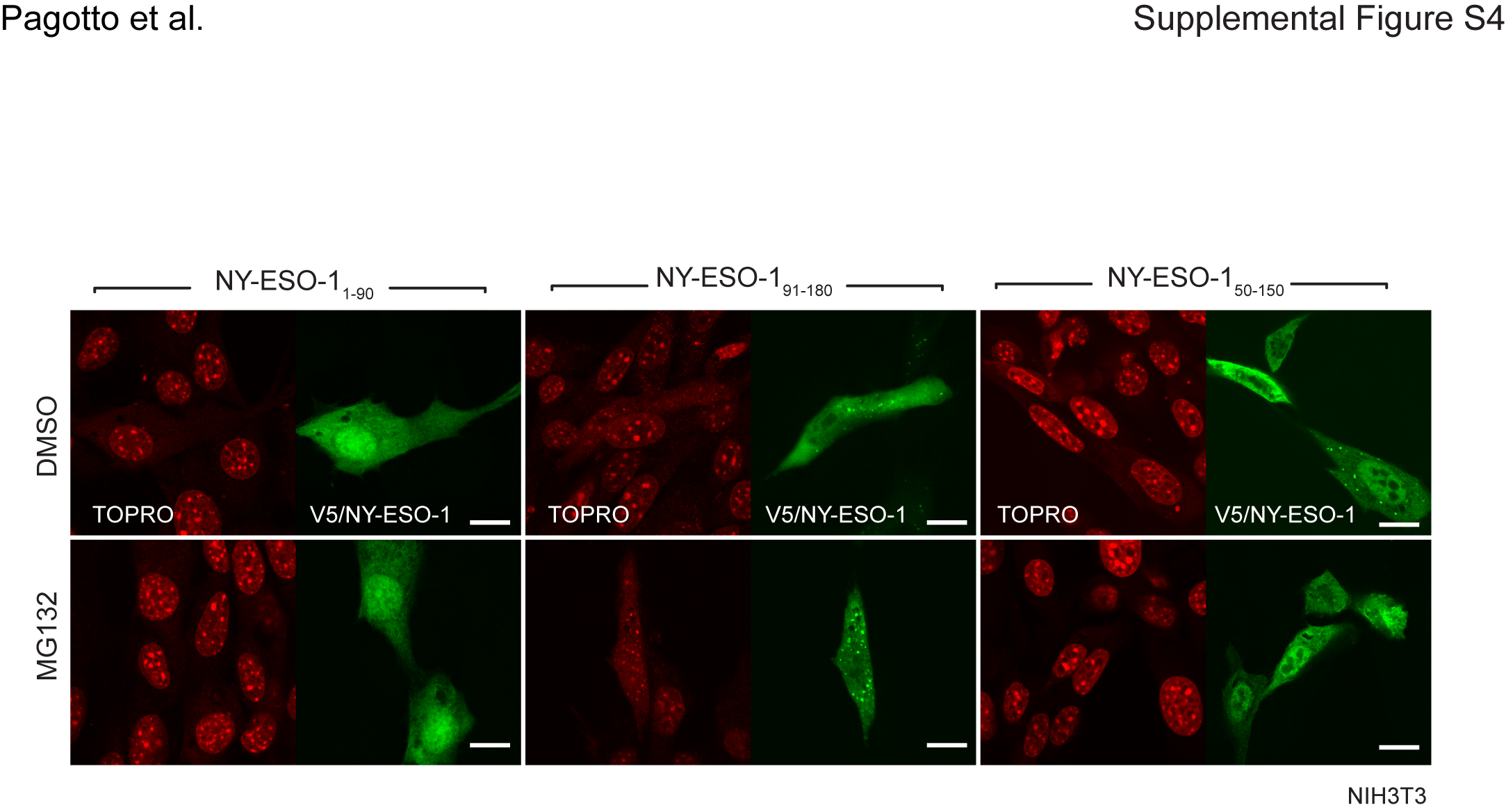

Supplement: Figure S4 — Localisation of transiently expressed NY-ESO-1 fragments in NIH3T3 cells. Immunofluorescence micrographs showing NIH3T3 mouse fibroblasts expressing NY-ESO-11-90, NY-ESO-191-180 and NY-ESO-150-150 (anti-V5, green). TOPRO was included to stain nuclei (red). Cells were treated with MG132 (40µM, 4hr) or DMSO (negative control). Scale bars = 20μm. (TIF) [file pone.0083212.s004.tif]

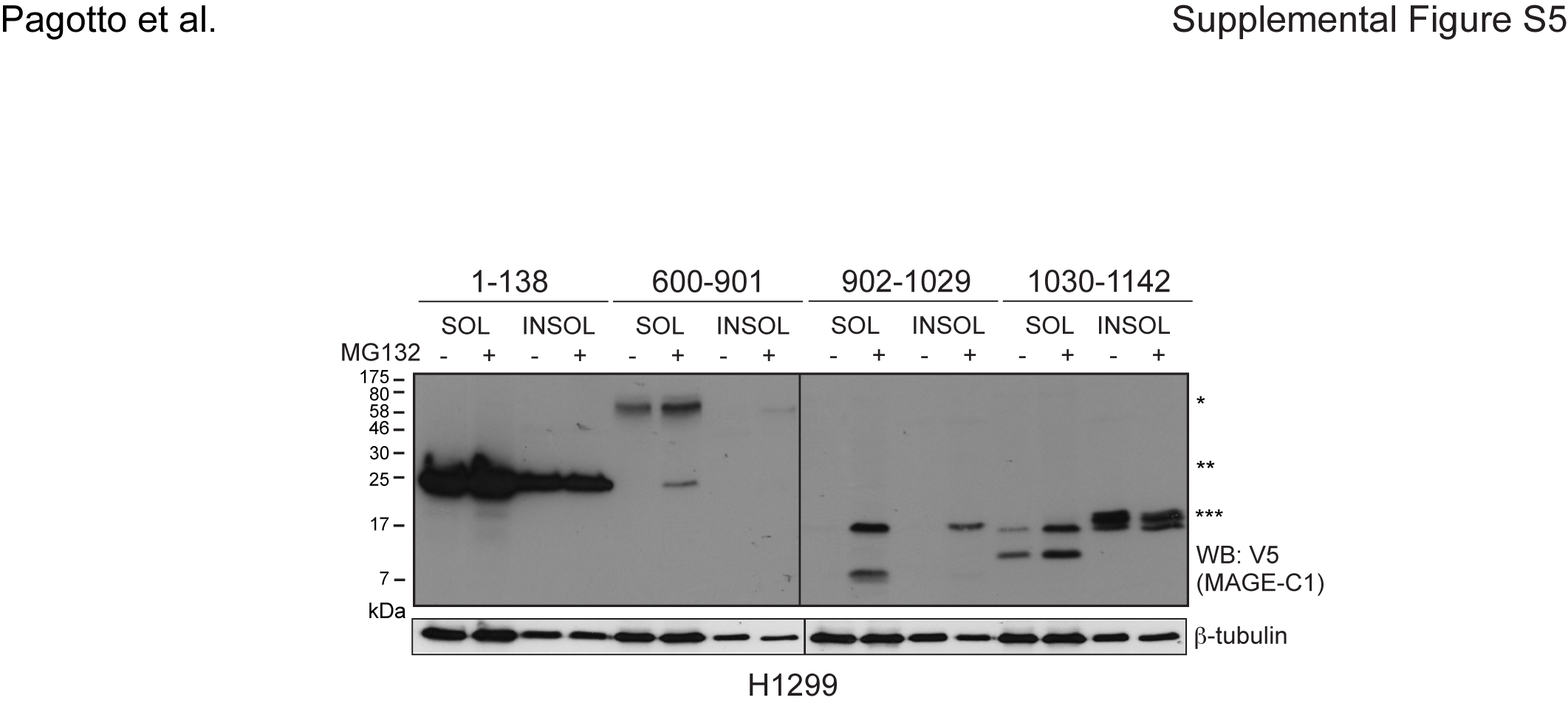

Supplement: Figure S5 — Solubility of MAGE-C1 fragments transiently expressed in H1299 cells. Transient expression of MAGE-C1 fragments in H1299 cells detected by western blot with anti-V5 and anti-β-tubulin (loading control). Cells were treated with DMSO and MG132 (40μM, 4hrs) and RIPA-soluble (SOL) and –insoluble (INSOL) fractions collected. Asterisks indicate possible oligomeric forms. (TIF) [file pone.0083212.s005.tif]
